# Continuous Positive Airway Pressure-Mandibular Advancement Device Combination Therapy for Moderate-to-Severe Obstructive Sleep Apnea: A Preliminary Study

**DOI:** 10.1055/s-0040-1719220

**Published:** 2021-01-07

**Authors:** Thyagaseely Sheela Premaraj, Jacob Stadiem, Shyamaly Arya Premaraj, Charles R. Davies, Matthew Dennis, John J. Harrington

**Affiliations:** 1 Department of Orthodontics, College of Dentistry, University of Nebraska Medical Center, Lincoln, Nebraska, United States; 2 Department of Orthodontics, University of Nebraska Medical Center, Lincoln, Nebraska, United States; 3 College of Dentistry, University of Nebraska Medical Center, Lincoln, Nebraska, United States; 4 Carle Neuroscience Institute, Carle Physician Group, University of Illinois at Urbana, Illinois, United States; 5 Division of Pediatric Pulmonology & Sleep Medicine, University of Nebraska Medical Center, Children’s Hospital & Medical Center, Omaha, Nebraska, United States; 6 Division of Pulmonary, Critical Care and Sleep Medicine, University of Nebraska Medical Center, Omaha, Nebraska, United States

**Keywords:** auto-adjusting positive airway pressure, Epworth Sleepiness Scale scores, mandibular advancement device, obstructive sleep apnea

## Abstract

**Objectives** The purpose of this pilot study was to determine whether compliance to auto-adjusting positive airway pressure (APAP) improves with the addition of a mandibular advancement device (MAD). Secondary outcome measures included were APAP pressure, subjective daytime sleepiness, apnea–hypopnea index (AHI), and mask leaks.

**Setting and Sample Population** Participants included were diagnosed with moderate-to-severe obstructive sleep apnea (OSA) and became noncompliant to prescribed APAP. Thirteen participants with a mean age of 61.6 years were recruited for this study.

**Materials and Methods** All participants were given a MAD to use with their APAP. Parameters measured included APAP pressure, AHI, mask leak reported via ResMed AirViewTM software, and self-reported daytime sleepiness (Epworth Sleepiness Scale [ESS]). A paired two-sample for mean *t* -test was performed to determine significance.

**Results** The mean difference of pre- and postintervention APAP compliance was 23.1%, which was statistically significant ( *p* = 0.015). The mean APAP air pressures were unchanged. The difference between pre- and postintervention mean ESS scores was 1.4 and was statistically significant ( *p* = 0.027). The mean difference between pre- and postintervention AHI values and mask leak showed no significant difference.

**Conclusion** This study showed that combination of APAP-MAD therapy, for patients with moderate-to-severe OSA who were noncompliant to APAP use, significantly increased compliance with APAP therapy, and significantly decreased the daytime sleepiness of participants.

## Introduction

The treatment of obstructive sleep apnea (OSA) allows for a multidisciplinary approach involving dentistry and medicine. The OSA results from collapse in the upper airway and leads to interruption of sleep as well as oxygen desaturations. 1 Patients suffering from OSA are at increased risk for myocardial infarction, heart failure, cerebrovascular insult, impaired cognition, and depression. 2

The treatment of OSA has not been a common practice in the dental profession until recently. The advent of mandibular advancement device (MAD) for the treatment of OSA introduced the dental field to sleep medicine. The MAD may be considered as an option for the initial treatment of mild-to-moderate OSA. 3 However, the MAD is also used as an alternative in patients exhibiting suboptimal compliance with continuous positive airway pressure (CPAP) therapy due to intolerance. 4 The most common complaints associated with CPAP include discomfort associated with the apparatus and intolerance to air pressure. 5 Improvements in CPAP machines and mask design have been made in an attempt to improve compliance. Despite potential side effects associated with the MAD, a preference for MAD therapy over CPAP for the treatment of OSA has been shown. 6

The MAD works by increasing the retropalatal and retro-lingual spaces while decreasing the length of the soft palate and angle of mouth opening. 7 One drawback of the MAD is its decreased effectiveness in improving AHI scores when compared with the CPAP. 8 One study showed a 42.8% AHI reduction with MAD therapy versus 73.2% with CPAP. 9 A review article on oral appliance treatment of sleep disordered breathing indicated that oral appliances successfully treat mild-to-moderate sleep apnea in 40 to 50% of patients and provide significant improvement in an additional 10 to 20%. 6

Clinicians are often faced with the dilemma of choosing between CPAP, which is highly effective but associated with suboptimal compliance, and MAD therapy that is less effective in severe AHI patients but associated with greater compliance. Suboptimal CPAP compliance undermines its effectiveness in treating OSA. The most critical time to establish long-term use patterns is often within the first week of treatment regardless of which therapy is prescribed. The extent to which a patient will use an appliance is determined in the first month of treatment. 10 Patients who experienced side effects or anxiety in the first 2 weeks of CPAP treatment were more likely to stop using the CPAP in the first year. 11 Also, patients who reported CPAP problems on the first night were less likely to maintain compliance. 12

While CPAP and MAD therapies have been in use for many years, it was only recently that a combination of the two treatments was studied. A pilot study, which looked at the efficacy of combination therapy, found that the combination treatment reduced the optimal CPAP pressure from 9.4 ± 2.3 to 7.3 ± 1.4 cm H _2_ O ( *p* < 0.001) compared with CPAP alone and decreased the apnea–hypopnea index (AHI) score from 11.2 ± 3.9 to 3.4 ± 1.5 per hour ( *p* < 0.001) compared with MAD alone. The subjects in the above study also tolerated the combination therapy very well. 13 Our present study is the first to evaluate the efficacy of combination therapy with subjects diagnosed with moderate to severe OSA. In addition, all subjects in the present study utilized APAP devices that also recorded nightly air pressure, duration of use, average AHI value, and mask leaks.

With the ongoing problem of suboptimal CPAP compliance limiting OSA treatment, the combination of CPAP-MAD therapy offers a promising alternative. This prospective study aims to improve the body of scientific knowledge in terms of efficacy and compliance of the APAP-MAD combination therapy. The objective of the study was to evaluate whether compliance with APAP could improve with the addition of a MAD. In addition, we evaluated the APAP pressure, subjective daytime sleepiness, AHI, and mask leaks pre- and postintervention with MAD.

## Materials and Methods

### Participant Acquisition

All participants were patients recruited from a sleep medicine clinic. After receiving institutional review board (IRB) approval (IRB: 017–16-EP), patients were recruited for this prospective study according to the inclusion and exclusion criteria. Inclusion criteria included diagnosis based on a sleep study indicating moderate-to-severe OSA as defined by an AHI score of ≥15 within the preceding 2 years, inability to tolerate CPAP-APAP or reported history of PAP noncompliance as defined by ≥4 hours per night for ≥30% of the nights over the preceding 3 months, presence of complete or functional dentition, age of 21 years and older, and auto-CPAP therapy using ResMed PAP device that is linked to ResMed AirView or has an SD card. Exclusion criteria included patients on oxygen therapy; history of alcohol abuse, narcotics, or daily sedating psychoactive medications; previous history of surgical treatments including uvulopalatopharyngoplasty or bilateral sagittal split osteotomy mandibular advancement; history of temporomandibular joint dysfunction, significant weight loss, or gain (10%) since diagnosis; history of claustrophobia or nasal airway obstruction or congestion (such as uncontrolled allergic rhinitis), other untreated sleep disorders (e.g., periodic limb movement disorder, restless legs syndrome, narcolepsy, central sleep apnea or insomnia); and history of congestive heart failure, chronic obstructive pulmonary disease, or psychiatric disorder other than controlled anxiety and/or depression. All of the criteria, other than functional dentition, were confirmed before the subjects were seen for the first study visit at the University of Nebraska School of Dentistry (UNMC) Adult Dental Clinic. Patients with partial or full dentures or any other oral appliances were excluded from the study. Based on the previously published pilot study, our power analysis showed that we needed 12 subjects to show significance in the change of pressure and compliance. 13

Each participant had previously been diagnosed with moderate-to-severe sleep apnea based on a diagnostic sleep study (i.e., home sleep apnea test or attended polysomnogram [PSG]) and had been prescribed APAP for at least 6 months. Suboptimal compliance with the APAP had been documented within 3 months prior to recruitment and was defined as using the device less than or equal to 4 hours a night for greater than or equal to 30% of the nights. Each subject had complete or functional dentition to support a MAD.

### Auto-Adjusting Positive Airway Pressure Protocol

All participants used a ResMed AirSense 10 AutoSet machine with the ability to automatically upload adherence data to ResMed AirViewTM (cloud-based system) or from an SD card for review. The patient’s machine was set to auto-adjusting positive airway pressure mode (APAP) at a pressure range of 6 to 20 cm H _2_ O. The adherence report consisted of information regarding the time of PAP usage, pressure requirements, residual respiratory events, and mask leak. The APAP machine adjusts the pressure automatically based on a proprietary algorithm. The data recovered from APAP machine included the participant’s hourly and daily usage, 95th percentile air pressure, AHI, and mask air leak. Participants were instructed to wear their APAP throughout the study. A person is considered compliant with the CPAP if they use CPAP ≥ 4 hours a night for at least 70% of the nights. 14 All measurements were taken 3 months prior to recruitment with the PAP only and over a 4-week period with combination therapy (APAP-MAD).

### Mandibular Advancement Device Protocol

**Fig. 1:**
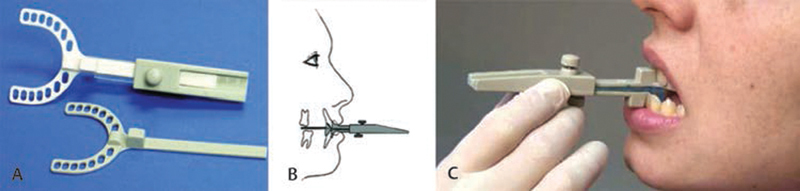
Measurement taking in mandibular advancement appliance fabrication. ( **A** ) George gauge with the bite forks. The white bite fork (top) has a 5-mm vertical opening in the anterior used in this study. The gray bite fork (bottom) has a 2-mm opening in the anterior. ( **B** ) Schematic of George gauge for measurement of mandibular range of movement. ( **C** ) George gauge being used to record a subject’s mandibular range of motion.

**Fig. 2:**
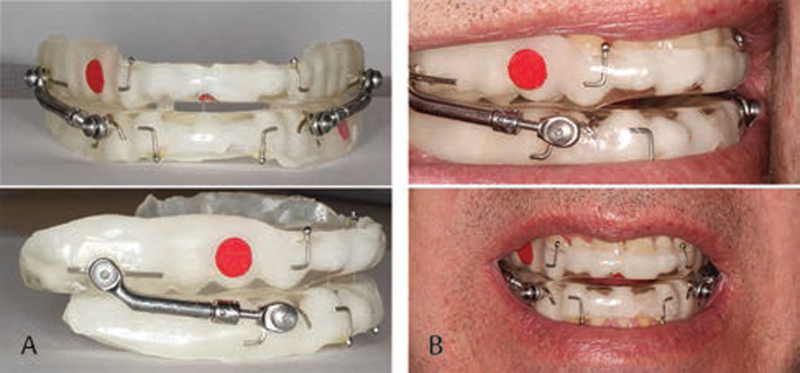
Herbst style MAD placement for forward mandibular position. ( **A** ) Herbst style mandibular advancement device. ( **B** ) Herbst style MAD in place. MAD, mandibular advancement device.

All participants reported to the UNMC Adult Dental Clinic to a single orthodontist. Impressions of the upper and lower teeth were taken using Identic 100-hour stability alginate, and models were poured up immediately in buff stone. A George Gauge ( Fig. 1A ) was used to determine where the bite registration was recorded ( Fig. 1B ). A 5-mm anterior bite fork was used for vertical opening ( Fig. 1C ). The initial bite registration was taken with the patients at 50% of their anteroposterior range of motion. The bite registration was taken using Exabite II NDS vinyl polysiloxane. The stone models and bite registration were sent to DynaFlex laboratories for the fabrication of an adjustable Herbst-design MAD ( Fig. 2A ). The MAD was made with a thermal acrylic material called AccuFit on the inside to allow for realignment when needed. At the second visit, the MAD was delivered, and participants were taught how to properly insert and remove the MAD. Upon delivery of the MAD, subjects were asked to wear the appliance for 2 weeks with their APAP and return to the UNMC Adult Dental Clinic for the evaluation of wear. Each participant was instructed to wear a 3/16-inch 3.5 oz elastic from upper to lower anterior hook bilaterally every night. After 2 weeks, participants were assessed for comfort with the appliance. If there was any temporo-mandibular joint discomfort, the appliance was modified to decrease the vertical dimension. Participants were then asked to wear the appliance for another 2 weeks and return for an assessment. Four participants needed vertical modification, but all of them were symptom free at the following visit. When subjects were free of discomfort, they were advanced to 70% of their anteroposterior range of motion ( Fig. 2B ). Participants were then instructed to wear the MAD with their APAP for 4 weeks. All data were recorded from the ResMed APAP machine.

### Self-Reported Assessment

Each participant completed an Epworth Sleepiness Scale (ESS) survey at their first appointment at the UNMC Adult Dental Clinic to assess their subjective daytime sleepiness. The ESS is a self-rated instrument ranging from 0 to 24, with scores greater than 10 denoting excessive daytime sleepiness. 15 After the final 4-week session of combination therapy, each subject completed another ESS survey. An increase in ESS score indicated an increase in daytime sleepiness, while a decrease in ESS score indicated a decrease in daytime sleepiness.

### Statistical Analysis

Means for 95th percentile pressure, compliance percentage, AHI value, and leaks were determined for preintervention (APAP only) and during postintervention (combination therapy). Mean ESS score for pre- and postintervention was also determined. A paired two-sample *t* -test was performed using SAS® (SAS Institute Inc., Cary, NC) to determine the significance of change in compliance, air pressure, AHI, mask air leaks, and ESS score. An unpaired two-sample *t* -test of equal variance was performed to determine the significance of the change in air pressure, adherence, and ESS score between males and females. An unpaired two-sample *t* -test of unequal variance was performed to determine the significance of the change in AHI and mask air leaks between males and females. An F-test was run for each data comparison between males and females to determine variance.

## Results

### Participant Acquisition

Forty-eight participants from a university-based sleep medicine clinic met the initial inclusion and exclusion criteria. After contacting potential participants, 18 participants showed interest in participating in this study. After initial recruitment, two participants did not have a functional dentition and three participants declined to participate. Thirteen participants started the study and all 13 completed the study.

### Demographics

The average age of the participants at the start of the treatment was 61.6 years with the range from 45.9 to 73.7 years. The gender makeup of the study was eight females (62%) and five males (38%). The average AHI score, before any treatment, of the participants was 40.6. Table 1 shows the pre- and postintervention mean values and the change in mean differences of all parameters recorded in this study with APAP alone and APAP-MAD therapy.

**Table 1:** Mean changes measured with auto-adjusting positive airway pressure alone versus auto-adjusting positive airway pressure-mandibular advancement device treatment for all participants

Subjects = 13						
	APAP alone mean	APAP- MAD mean	Mean difference	SD	*p* -Value	Significance
Pressure (cm H _2_ O)	12.4	12.2	−0.2	1.7	0.696	NS
Compliance percentage	44.3	67.4	23.1	29.5	0.015	^a^
ESS	7.6	6.2	−1.4	2.0	0.027	^a^
AHI	2.1	2.0	−0.1	2.1	0.908	NS
Mask air leaks (L/min)	15.2	14.3	−0.9	9.6	0.754	NS

Abbreviations: AHI, Apnea–Hypopnea Index; APAP, adjusting positive airway pressure; ESS, Epworth Sleepiness Scale; MAD, mandibular advancement device; NS, nonsignificance; SD, standard deviation.

a Indicate statistical significance.

### Compliance Percentages

**Fig. 3:**
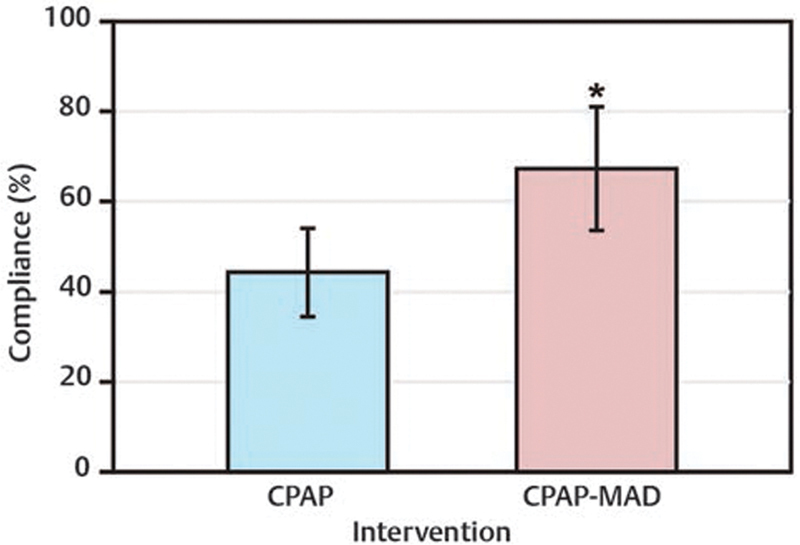
Continuous positive airway pressure compliance before and after combination therapy. Pre- and postintervention mean compliance percentage. Statistically significant increase in compliance percentage observed (* *p* = 0.015).

The average pre- and postintervention for percentage compliance of APAP use for all participants are shown in Fig. 3 . Error bars represent the upper and lower 95% confidence limits of each measurement. The *p* -value for all compliance percentages was set at <0.05. Nine out of 13 participants (69%) showed an increase in adherence percentage, three out of 13 participants (23%) exhibited a decrease in adherence percentage, and one out of 13 participants (8%) exhibited no change in adherence percentage. An average increase in adherence percentage of 23.1% with combination therapy was statistically significant ( *p* = 0.015).

### Epworth Sleepiness Scale

**Fig. 4:**
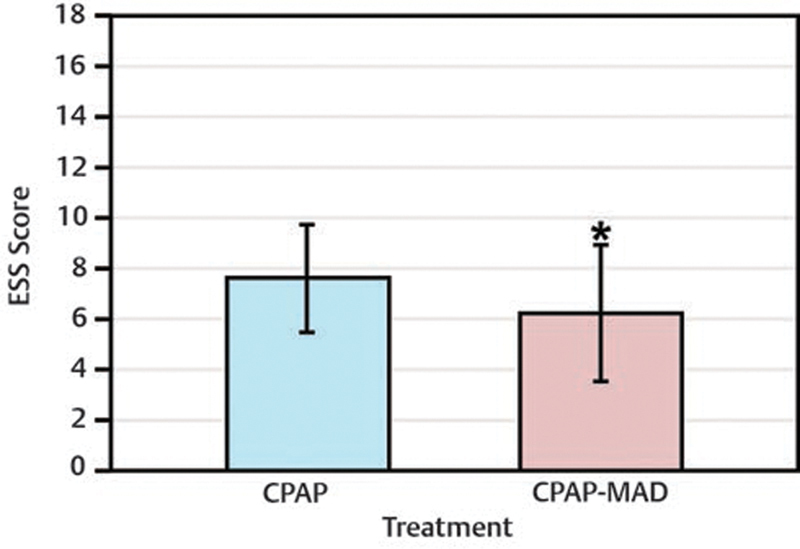
Pre and postintervention ESS scores. Pre and postintervention mean ESS scores. Statistically significant decrease in ESS scores observed (* *p* = 0.027). ESS, Epworth Sleepiness Scale.

The average pre- and postintervention ESS scores for all participants are shown in Fig. 4 . Error bars represent the upper and lower 95% confidence limits of each measurement. The *p* -value for all ESS scores was set at <0.05. Nine out of 13 participants (69%) showed a decrease in their ESS score, three out of 13 participants (23%) exhibited an increase in their ESS score, and one out of 13 participants (8%) exhibited no change in their ESS score. An average decrease in ESS score of 1.4 with combination therapy was statistically significant ( *p* = 0.027).

### Apnea–Hypopnea Index Values

**Fig. 5:**
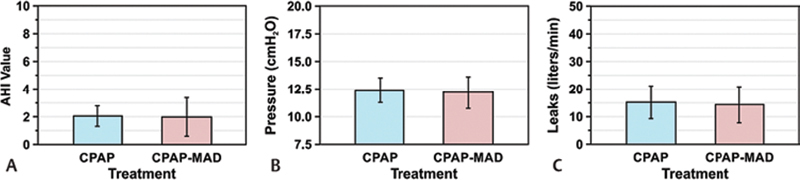
Pre and postintervention AHI, air pressure, and mask air leaks measurements. ( **A** ) Pre and postintervention mean AHI scores. AHI did not show any significant changes with intervention. ( **B** ) Pre- and postintervention mean 95th percentile air pressure. CPAP air pressures were measured 3 months prior to recruitment with the CPAP only and over a 4-week period with intervention therapy. No significant changes observed. ( **C** ) Pre- and postintervention mask air leaks did not show any significant differences. AHI, Apnea–Hypopnea Index; CPAP, continuous positive airway pressure.

The average pre- and postintervention AHI values for all participants are shown in Fig. 5A . Error bars represent the upper and lower 95% confidence limits of each measurement. The *p* -value for all AHI values was set at <0.05. Nine out of 13 participants (69%) showed a slight decrease in their AHI value, still within their therapeutic values set by the physicians during sleep study. Three out of 13 participants (23%) exhibited a slight increase in their AHI value and again maintained within the therapeutic values. One out of 13 participants (8%) exhibited no change in their AHI value. An average decrease in AHI value of 0.7 was not statistically significant ( *p* = 0.908).

### Auto-Adjusting Positive Airway Pressure Air Pressure Levels

The average pre- and postintervention measurements for APAP air pressure on all participants are shown in Fig. 5B . Error bars represent the upper and lower 95% confidence limits of each measurement. The *p* -value for all pressure measurements was set at <0.05. Six out of 13 participants (46%) showed a decrease in pressure, while seven out of 13 (54%) exhibited an increase in pressure used with the APAP. An average decrease in pressure of 0.2 cm H _2_ O was not statistically significant ( *p* = 0.697).

### Mask Leak with Auto-Adjusting Positive Airway Pressure

The average pre- and postintervention measurements for APAP mask leak on all participants are shown in Fig. 5C . Error bars represent the upper and lower 95% confidence limits of each measurement. The *p* -value for all leak measurements was set at <0.05. Six out of 13 participants (46%) showed a decrease in leak, while seven out of 13 (54%) exhibited an increase in leak while using the combination therapy. An average decrease in leak of 0.9 L/min was not statistically significant ( *p* = 0.754).

## Discussion

The gold standard for OSA treatment is CPAP therapy. However, CPAP is associated with high nonadherence rates. Adherence with CPAP therapy studied for both short-term (within months after initiation) and long-term use showed less than 50% adherence. 16 The main obstacles in achieving CPAP adherence are related to side effects, such as increased awakenings and dry mouth. 11 Another common factor is aerophagia, which causes discomfort during and after CPAP use. The goal of our study was to determine if CPAP adherence can improve with the addition of a MAD. The rationale of this study is twofold. First, a MAD is preferred as an alternative to CPAP by many patients. 6 Second, MAD therapy increases the volume of upper airway 17 and may therefore reduce overall CPAP pressure requirements to maintain airway patency. This reduced airflow pressure may improve tolerance to PAP and increase time of use. When MAD failed to improve comfort level, participants were likely to remain noncompliant.

This study evaluated compliance with APAP when MAD was utilized as an adjunctive therapy to determine whether an APAP-MAD combination treatment could improve PAP adherence when noncompliance was identified before enrollment. Wolkove et al demonstrated that long-term CPAP adherence was related to initial experiences with CPAP therapy. 16 In this study, patients were already APAP therapy and their average usage was measured through ResMed AirView/SD card downloads, and was calculated as the percentage of 30 days the subject wore the CPAP ≥4 hours per night. We recorded PAP compliance during combination therapy for 1 month after the final adjustment of the MAD. Our study showed a statistically significant increase in compliance percentage scores ( *p* = 0.015), indicating better compliance with the combination therapy. The average percentage of PAP use in our study with the APAP-MAD combination therapy was 67.4%. Although the average percentage of use was slightly under 70%, six out of 13 participants did achieve this threshold. Nine out of 13 participants showed an increase in compliance percentage, with three subjects exhibiting a 97% compliance percentage. However, long-term assessment of compliance with combination therapy would be important to determine the effectiveness and tolerability of this combination treatment.

APAP pressure levels were measured remotely, and each PAP device automatically adjusted delivered pressures to maintain airway patency. The autotitrating PAP was set by the sleep medicine providers at a pressure range of 6 to 20 cm H _2_ O. The average pressure used with the APAP therapy alone was 12.4 cm H _2_ O. With the combination treatment, the average pressure recorded was 12.2 cm H _2_ O, indicating the decrease was not statistically significant ( *p* = 0.697). Our results vary from the pilot study reported by El-Solh et al who found a significant difference in air pressure requirement with the combination therapy. 13 They compared the APAP-MAD combination therapy to fixed pressure CPAP therapy, and their study was conducted over only through three consecutive nights. A meta-analysis showed that APAP pressure recorded was on average was 2.2 cm H _2_ O less than fixed CPAP delivery. 18 This may have influenced the results of their study. Further, they included participants with mild, moderate, and severe OSA, and their subjects had much lower starting CPAP pressures than our current study.

The present study indicated a significant increase in compliance with the combination therapy even though the mean air pressures did not change significantly. Although PAP pressure intolerance has been regarded as a reason for nonadherence to CPAP therapy, our study revealed increase PAP adherence without any statistically significant change in mean pressure requirements.

During use of a MAD, the mandible is positioned forward and downward. All participants in this study were advanced to a 70% of the maximum anteroposterior range of the lower jaw. The change in morphology of the oropharynx as a result of the forward and downward jaw position may lead to changes in airflow dynamics with less turbulent flow, reduced aerophagia, and a change in pharyngeal critical pressure to reduce discomfort.

The ESS survey was administered during the MAD fabrication visit and again at the end of the combination therapy. Participants in our study were subjectively less sleepy with combination therapy compared with APAP alone. The average ESS score with APAP therapy alone was 7.6, and the average ESS score after combination therapy was 6.23, a statistically significant mean difference of 1.4 ( *p* = 0.027). The ESS test-retest reliability has been shown to be reliable with no statistically significant changes when there is no treatment between the first and second time the test is administered. 15 The ESS has also been shown to be reliable in reporting a reduction of day-time sleepiness with OSA treatment. 19 Improvements in self-reported sleepiness may be related to increased adherence with combination therapy (APAP-MAD).

All participants in our study had mean pretreatment AHI scores of 40.6 (20.1–93.8) and had residual scores of <5 per hour with APAP alone. The average AHI score with the APAP alone was 2.1 and with the combination therapy was 2.0, and this difference was not statistically significant. The AHI scores for this study with combination therapy were derived from PAP compliance downloads and not an attended PAP titration PSG. However, a study by Desai et al showed that the estimate of residual AHI by an APAP compliance downloads showed good agreement with the AHI determined from PSG. 20 As all subjects’ prescribed PAP pressures were determined to adequately control sleep apnea prior to study enrollment, there was no intention to further decrease residual AHI values. The combination therapy maintained therapeutic levels of AHI.

One potential problem with adding MAD to existing PAP therapy is the probability that the mask may not fit properly. Ideally, the participant should be fit for a new mask with the appliance in place to ensure the appliance is not causing the mask to leak. In this study, the participants continued wearing their existing APAP mask. The average preintervention mask leak was 15.2 L/min and decreased to 14.4 L/min with combination therapy, but this difference was not statistically significant ( *p* = 0.754). Mouth opening, mean APAP pressure, sleep position, and rapid eye movement sleep have been shown to be factors that increase unintentional mask leakage. 21 Mask air leak with oral dryness or nasal congestion has been associated with poor adherence to CPAP. 22 23 The use of elastics with the appliance to keep the subject’s mouth from opening in this study may have helped avoid unwanted leaks in our study. However, while air leaks did not decrease in this study, they also did not worsen.

There were several limitations to this present study, including reduced sample size possibly related to difficulty in recruiting subjects due to their lack of knowledge regarding oral appliance therapy and its potential effectiveness in improving sleep-related problems. Gender differences in compliance could not be assessed due to our small sample size. There was no control group for comparison and APAP-MAD compliance was compared with APAP 30-day compliance downloads collected prior to enrollment.

MAD improved the usage of PAP therapy in the treatment of OSA in this study. However, there were individuals who did not respond positively to APAP-MAD combination therapy demonstrated by increased AHI values or ESS scores. To evaluate why some individuals respond well to treatment while others do not, it may be informative to assess airway dimensional changes with three-dimensional imaging techniques with various forms of therapy. In addition, analyzing the airflow pattern changes utilizing computational fluid mechanics could provide a better understanding of the underlying physiology of sleep apnea and effects of treatment.

Despite the described limitations, this study provides some evidence that combination therapy and a multidisciplinary approach can positively affect OSA compliance. Future randomized clinical controlled trials of combination therapy with increased sample sizes would be helpful to confirm the effectiveness of this treatment approach. The inclusion of control (sham oral appliances) could be considered.

## Conclusion

Poor CPAP compliance is a major clinical issue for patients and their medical providers. The most common reason for failing CPAP therapy is discomfort during the initial treatment period along with difficulty initiating or maintaining sleep. 16 This pilot study demonstrated that APAP-MAD combination therapy significantly increased PAP compliance and significantly improved subjective sleepiness. Larger randomized controlled studies are needed to confirm these findings.
